# Association of HLA‐B Leader With BK Polyomavirus and Cytomegalovirus Infections in Kidney Allograft Recipients

**DOI:** 10.1111/tan.71082

**Published:** 2026-11-06

**Authors:** Leonie Ballmer, Matthias Diebold, Hans H. Hirsch, Gideon Hönger, Caroline Wehmeier, Stefan Schaub

**Affiliations:** ^1^ Clinic for Transplantation Immunology and Nephrology University Hospital Basel Basel Switzerland; ^2^ Translational Virus Immunity Pathology, Immunology Research, Department of Medical Biology UiT The Arctic University of Norway Tromsø Norway; ^3^ Translational Virus Immunity Pathology, Institute of Experimental Immunology University of Zurich Zurich Switzerland; ^4^ HLA‐Diagnostics and Immunogenetics, Department of Laboratory Medicine University Hospital Basel Basel Switzerland

**Keywords:** BK polyomavirus, cytomegalovirus, HLA‐B leader, kidney transplantation

## Abstract

Natural killer (NK) cell education and function depend on HLA‐E expression, influenced by the dimorphic HLA‐B leader peptide (−21M vs. −21T). NK cells help control BK polyomavirus (BKPyV) and cytomegalovirus (CMV) after kidney transplantation, but the HLA‐B leader's role is unclear. We retrospectively studied 700 kidney transplants with high‐resolution recipient/donor HLA typing, determining the recipient's HLA‐B leader genotype (MM 6%, MT 39%, TT 55%) and recipient/donor constellation (345/700 [49%] matched, 355/700 [51%] mismatched). Associations with 1‐year occurrence and severity of BKPyV (decoy cell shedding, BKPyV‐DNAemia, presumptive BKPyV‐associated nephropathy [BKPyVAN]) and CMV (DNAemia, disease) were assessed with univariate analyses and multivariable Cox and logistic regression. One‐year incidence of decoy cell shedding was similar across genotypes (MM 28.3%, MT 29.0%, TT 33.5%; *p* = 0.37) and between matched and mismatched recipients (31.0% vs. 31.8%; *p* = 0.79); BKPyV‐DNAemia incidence likewise did not differ by genotype (MM 19.6%, MT 16.5%, TT 18.9%; *p* = 0.73) or constellation (15.9% vs. 20.0%; *p* = 0.16). In multivariable models, neither genotype nor constellation was associated with decoy cell shedding, BKPyV‐DNAemia, or development of presumptive BKPyVAN. CMV‐DNAemia incidence also did not differ by genotype (MM 19.6%, MT 30.9%, TT 28.8%; *p* = 0.35) or constellation (27.0% vs. 31.0%; *p* = 0.25). In multivariable models, neither genotype nor constellation was associated with CMV‐DNAemia or development of CMV disease. In conclusion, the HLA‐B leader is neither associated with the occurrence nor the severity of BKPyV and CMV infections within the first year posttransplant in kidney allograft recipients. This argues against using HLA‐B leader information to guide BKPyV or CMV screening strategies.

**Abbreviations**
BKPyV: BK polyomavirus
BKPyVAN: BK polyomavirus‐associated Nephropathy
CI: confidence interval
CMV: cytomegalovirus
HR: hazard ratio
MPA: mycophenolic acid
NK cells: natural killer cells
OR: odds ratio
PCR: polymerase chain reaction
Tac‐MPA‐P: tacrolimus, mycophenolic acid and prednisone

## 1 Introduction

Cytomegalovirus (CMV) and BK Polyomavirus (BKPyV) infections are common after kidney transplantation and can cause significant complications including graft loss and patient death [1, 2, 3, 4, 5, 6]. Therefore, identification of risk factors for CMV and/or BKPyV infections available already pretransplant may help to guide screening protocols and prophylactic measures.

For CMV, the pretransplant serostatus between the donor (D) and recipient (R) can be categorised into three groups: low risk (R−/D−), intermediate risk (R+/D− or R+/D+) and high risk (R−/D+). These risk categories guide decisions on screening and antiviral prophylaxis [7, 8]. For BKPyV, several risk factors such as tacrolimus‐based maintenance immunosuppression, older age and male sex have been reported [9]. In addition, some HLA alleles of the recipient were found to be protective in larger studies [9, 10]. Among these are HLA‐B7, HLA‐B8 and HLA‐B51, which can particularly well present the BKPyV T antigen‐derived 9mer LPLMRKAYL to CD8+ cytotoxic T‐cells [11, 12, 13, 14]. However, the current BKPyV‐associated risk factors are not regarded as significant enough to guide current management strategies for BKPyV infection [9].

Besides CD8+ cytotoxic T‐cells, natural killer cells (NK cells) play an important role in viral infection control [15]. NK cell education and function are shaped in part by the inhibitory receptor NKG2A, which engages HLA‐E [16]. HLA‐E expression on cell surfaces itself depends on stable binding of HLA leader sequence derived peptides [17]. The HLA‐B leader sequence is dimorphic, exhibiting either a methionine (M) or threonine (T) at position −21. It has been shown that −21M leader derived peptides upregulate HLA‐E expression more effectively than −21T leader‐derived peptides and hence promote NK cell education and function [16, 18]. While the HLA‐B leader of the recipient might be important for control of any viral infection, the situation for BKPyV is likely more complex and could also depend on the HLA‐B leader constellation between the recipient and the donor, because BKPyV primarily replicates in the kidney allograft. Guberina et al. [19] reported that kidney allograft rejection was associated with higher numbers of HLA Class I leader peptide mismatches and higher HLA‐E expression in the allograft. Therefore, HLA‐B leader mismatch could potentially not only lead to a stronger immune response against alloantigens, but possibly also to a better control of BKPyV in the kidney transplant.

In 2025, Aubry et al. [20] were the first to explore the association of the HLA‐B leader constellation with the occurrence of CMV and BKPyV infection after kidney transplantation. In 306 kidney allograft recipients they found that a recipient/donor HLA‐B leader mismatch was associated with a 52% reduced risk for BKPyV‐DNAemia or BKPyV‐DNAuria > 7 log_10_ copies/mL within the first year posttransplant. By contrast, no such association was found for CMV‐DNAemia. Limitations of the study were a rather small population size, lack of high‐resolution HLA typing of the donors to determine the HLA‐B leader sequences and absence of granular description of CMV and BKPyV infection phenotypes. The authors acknowledged that their intriguing observation requires validation in larger independent cohorts [20].

The aim of this retrospective analysis was to independently validate and extend the above findings by investigating the association between HLA‐B leader determined by high‐resolution HLA typing and the occurrence as well as the severity of BKPyV and CMV infection in 700 well‐characterised kidney allograft recipients.

## 2 Methods and Materials

### 2.1 Patient Selection and Description of Study Population

This retrospective study was approved by the local ethics committee of North‐Western and Central Switzerland (EKNZ 2026–00962). All patients who received a kidney allograft at the University Hospital Basel between January 2014 and December 2024 were eligible for the study (*n* = 848). Exclusion criteria were defined as follows: graft loss or death within the first year after transplantation, decline of institutional general consent, not having received induction therapy, patients with a maintenance immunosuppression regimen that differed from the standard regimen with tacrolimus (Tac)–mycophenolic acid (MPA)–prednisone (P) or missing data (no high‐resolution HLA‐typing, no CMV and/or BKPyV screening, undetermined HLA‐B leader genotype). Of the 848 patients, 148 (17%) had to be excluded, leaving 700 patients for the analysis (Figure 1).

**FIGURE 1:**
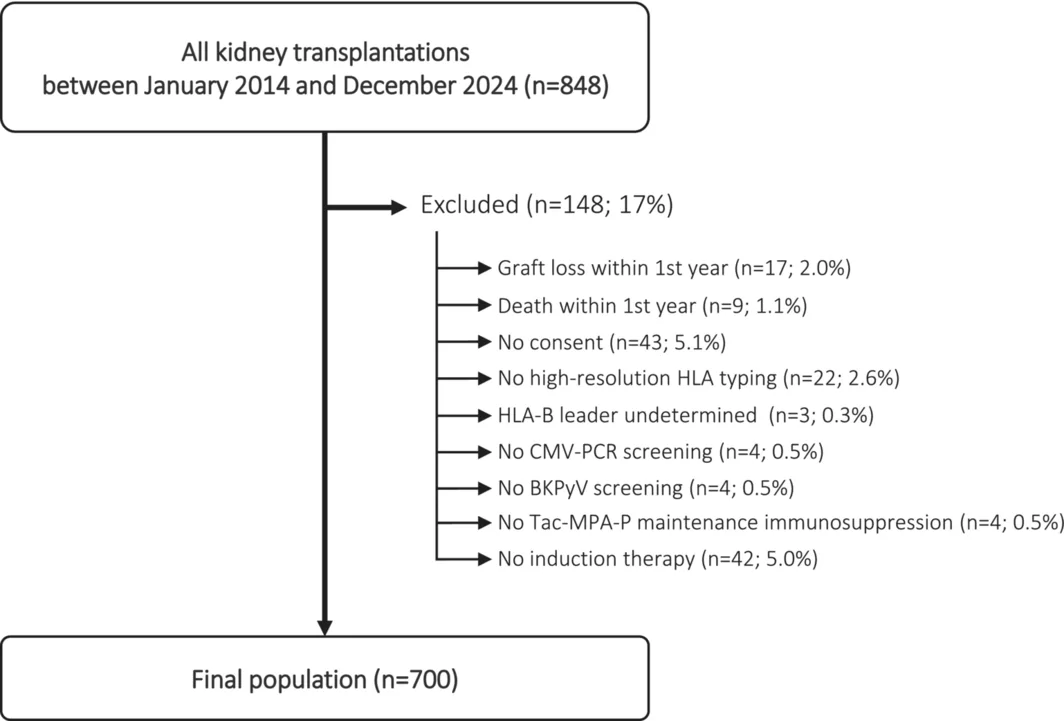
Flowchart of the study population. MPA, mycophenolic acid; Pred, prednisone; Tac, tacrolimus.

### 2.2 Screening for BKPyV Infection and Its Management

Screening for BKPyV and its management at our institution have been reported previously [21]. Briefly, patients underwent screening for BKPyV at regular intervals by analysis of urine decoy cells (every 2 weeks during the first 3 months, monthly thereafter until month six, and then again at 12 months). Additional screening analyses were performed in case of clinical suspicion. Decoy cells are mostly tubular epithelial cells released into the urine, which contain large amounts of BKPyV. A BKPyV‐DNAuria > 10^6^ GEq/mL is strongly correlated with decoy cell shedding in urine [9]. If decoy cells were detected, BKPyV‐DNA loads were determined in plasma by quantitative nucleic acid testing (QNAT). BKPyV‐DNAemia > 1000 GEq/mL was considered positive. If persisting BKPyV‐DNAemia was detected, immunosuppression was decreased gradually and BKPyV‐DNAemia monitored. If decoy cells were detected without BKPyV‐DNAemia, immunosuppression was not adapted, but decoy cell monitoring intensified.

### 2.3 Screening for CMV Infection and Its Management

Screening and management of CMV at our institution has been previously described [8]. Briefly, according to international guidelines, a pretransplant risk stratification was performed with respect to recipient and donor CMV serostatus [7]. Patients were categorised into high‐ (D+/R−), intermediate‐ (R+) and low‐ (D−/R−) risk groups. Patients with high‐risk serostatus or anti‐thymocyte globulin (ATG) induction combined with an intermediate‐risk serostatus received Valganciclovir prophylaxis for 3 months. If immunosuppression at 3 months was still considered as high, prophylaxis was extended up to 6 months. After completion of prophylaxis, screening for CMV‐DNAemia was started every other week for 3 months. Intermediate‐risk patients, who did not receive ATG induction were screened for CMV‐DNAemia starting 2 weeks after transplantation at weekly intervals for the first 3 months, then every 2 weeks until month six. Patients with low‐risk serostatus received neither prophylaxis nor screening for CMV‐DNAemia. Additional testing for CMV‐DNAemia beyond the screening protocol was performed in all patients based on clinical suspicion. CMV QNAT results > 137 IU/mL (until 09/2022) or > 34.5 IU/mL (after 09/2022 due to change of the assay) were considered positive.

### 2.4 Definition of BKPyV and CMV Infection Phenotypes

For defining BKPyV infection the following categories were used according to international consensus guidelines [9]: ‘No decoy cells’ if decoy cells were undetectable; ‘Decoy cells, no BKPyV‐DNAemia’ if decoy cells were detectable, but plasma BKPyV‐DNAemia was undetectable; ‘Low BKPyV‐DNAemia’ if plasma BKPyV‐DNA loads were detectable but below 10′000 GEq/mL; ‘High BKPyV‐DNAemia’ if BKPyV‐DNA loads exceeded 10′000 GEq/mL (also referred to as presumptive BKPyV‐associated nephropathy [BKPyVAN]). The category ‘biopsy‐proven/definitive BKPyVAN’ was not used as an endpoint to avoid bias, because allograft biopsies are not routinely performed for the diagnosis of BKPyVAN in our institution [21].

CMV infection was also categorised based on international consensus definitions as follows [22]: ‘No CMV‐DNAemia’ if screening CMV DNA was undetectable; ‘Asymptomatic CMV‐DNAemia’ if CMV DNA was detectable but corresponding clinical signs or symptoms were lacking; ‘CMV disease’ for patients with CMV‐DNAemia and corresponding clinical signs or symptoms. ‘CMV disease’ was further divided into ‘CMV syndrome’ and ‘Tissue‐invasive CMV disease’. ‘CMV syndrome’ was defined as detectable CMV DNA and at least two further criteria, such as fever, malaise, leukopenia, atypical lymphocytes, thrombocytopenia and/or elevation of hepatic transaminases. In ‘Tissue‐invasive CMV disease’, symptoms corresponding with end‐organ involvement, for example gastrointestinal disease or pneumonia, had to be present along with CMV‐DNAemia.

### 2.5 Determination of HLA‐B Leader and Grouping

High‐resolution HLA‐typing including all 11 loci was performed by next‐generation sequencing using the NGSgoMX11‐3 kits and workflow from GenDx (gendx.com) for all included donors and recipients as reported previously [23]. The amino acid at position −21 of the HLA‐B leader peptide was determined by sequence information of the IMGT‐database (version 3.62). The HLA‐B leader of the recipients alone (i.e., MM, MT, TT) or the HLA‐B leader constellation between the recipient and the donor were used to create patient groups.

### 2.6 Data Collection and Statistical Analysis

We collected clinical data by investigating each patient's medical records, which were stored either in hard copy in our archive or electronically in our hospital's clinical information system. Data were collected from the day of transplantation up to the follow‐up appointment 1 year after transplantation.

Statistical analyses were primarily performed using JMP version 19.1.1 (SAS Institute, Cary, NC). R Version 4.6.0 (R Foundation for Statistical Computing, Vienna, Austria) was used to assess Schoenfeld residuals, stratify Cox proportional hazards models for variables violating the proportional hazards assumption and verify the results obtained with JMP. A two‐tailed *p*‐value < 0.05 was deemed statistically significant. None of the variables were normally distributed, as assessed by Q–Q plots and the Shapiro–Wilk test. For numerical data, medians and interquartile ranges (IQRs) are reported, and differences assessed by Wilcoxon rank‐sum test. Counts and percentages were used to describe categorical data, and differences assessed by Pearson Chi‐squared test (*χ* ^2^). Time‐to‐event data were visualised with Kaplan–Meier curves and analysed with a log‐rank test. To analyse whether HLA‐B leader status was independently associated with time to occurrence of viral infection, we fitted three separate multivariable Cox regression models for time to decoy cell shedding, BKPyV‐DNAemia (i.e., BKV PCR > 1000 GEq/mL) and CMV‐DNAemia (i.e., PCR > 137 IU/mL or > 34.5 IU/mL, respectively). For the first two BKPyV models, we adjusted for factors that are established risk factors of BKPyV infection in kidney allograft recipients and/or that showed a significant difference between the HLA‐B leader groups in the univariate analysis (i.e., recipient age, recipient sex, induction therapy, HLA mismatches, recipient HLA‐B7‐, HLA‐B8‐ or HLA‐B51‐positive) [9]. For the CMV model we additionally included CMV serostatus and prophylaxis with Valganciclovir as predictors of CMV‐DNAemia, but did not include recipient sex and induction therapy as they have not been reported as risk factors of CMV infection in the past [24, 25]. There were no missing data in the included covariates. The proportional hazards assumption was assessed visually using scaled Schoenfeld residual plots and formally using Schoenfeld residual tests. There was a minor violation of the proportional hazards assumption for induction therapy and CMV risk group; however, the scaled Schoenfeld residual plot showed no systematic trend over time. The effect of CMV prophylaxis violated the proportional hazards assumption and was stratified in the final Cox regression model. Finally, to investigate the independent association of the HLA‐B leader with the most severe CMV and BKPyV infection phenotype (i.e., CMV disease and high BKPyV‐DNAemia), we fitted two binary logistic regressions, adjusting for the same factors as described above.

## 3 Results

### 3.1 Baseline Characteristics According to Recipient HLA‐B Leader

Baseline characteristics of the whole study population are summarised in the first two columns of Table 1. In the whole population, the median recipient age was 55 years (IQR 44–63) and 34% were female. The median donor age was 56 years (IQR 47–64) and 41% of transplantations were from a living donor. Patients had induction therapy with either ATG (18%) or Basiliximab (82%).

**TABLE 1:** Baseline characteristics of patients according to HLA‐B leader constellation between donor and recipient.

Variable	Whole population (*n* = 700)	HLA‐B leader matched (*n* = 345)	HLA‐B leader mismatched (*n* = 355)	*p*
Recipient age (years)	55 (44–63)	52 (42–62)	57 (47–65)	< 0.001
Recipient female, *n* (%)	240 (34)	128 (37)	112 (32)	0.12
Underlying nephropathy, *n* (%)				0.53
Glomerulopathy	234 (33)	121 (35)	113 (32)	
Vascular/hypertensive	67 (10)	26 (8)	41 (12)	
Diabetic	55 (8)	27 (8)	28 (8)	
ADPKD	144 (21)	68 (20)	76 (21)	
Interstitial	38 (5)	21 (6)	17 (5)	
Unknown	64 (9)	35 (10)	29 (8)	
Other	98 (14)	47 (14)	51 (14)	
Induction therapy, *n* (%)				0.60
ATG	123 (18)	58 (17)	65 (18)	
Basiliximab	577 (82)	287 (83)	290 (82)	
Living donor, *n* (%)	290 (41)	152 (44)	138 (39)	0.16
Donor age (years)	56 (47–64)	56 (47–63)	57 (47–64)	0.44
First kidney transplant, *n* (%)	605 (86)	302 (88)	303 (85)	0.40
CMV serostatus, *n* (%)				0.52
Low risk (D−/R−)	170 (24)	79 (23)	91 (26)	
Intermediate risk (R+)	409 (58)	209 (61)	200 (56)	
High risk (D+/R−)	121 (17)	57 (17)	64 (18)	
CMV prophylaxis with Valganciclovir, *n* (%)	243 (35)	118 (34)	125 (35)	0.78
Pretransplant HLA‐DSA, *n* (%)	91 (13)	39 (11)	52 (15)	0.19
HLA‐A/B/C/DRB1/DRB3‐5/DQB1/DPB1 mismatches^a^	8 (6–10)	7 (5–9)	8 (6–10)	< 0.001
Recipient HLA‐B7, HLA‐B8 or HLA‐B51 positive, *n* (%)	338 (48)	144 (42)	194 (55)	< 0.001
ABO incompatible, *n* (%)	55 (8)	31 (9)	24 (7)	0.27
Delayed graft function, *n* (%)	155 (22)	69 (20)	86 (24)	0.18

Abbreviations: ADPKD, autosomal dominant polycystic kidney disease; ATG, anti‐thymocyte globulin; CMV, cytomegalovirus; HLA‐DSA, donor‐specific HLA‐antibodies.

^a^ HLA mismatches are calculated at the level of serological equivalents.

The most frequent HLA‐B leader was TT (*n* = 382; 55%) followed by MT (*n* = 272; 39%) and MM (*n* = 46; 6%). Therefore, 318 recipients (45%) had at least one −21M peptide, 382 recipients (55%) had no −21M peptide. The baseline characteristics stratified by the presence/absence of a −21M peptide are detailed in Supplementary Table 1. Recipients with a −21M peptide received slightly more often CMV prophylaxis with Valganciclovir (39% vs. 31%; *p* = 0.04), had fewer HLA mismatches (median 7 vs. 8; *p* = 0.02) and had more often HLA‐B7, −B8 or −B51 alleles (78% vs. 23%; *p* < 0.001).

### 3.2 Baseline Characteristics According to Recipient/Donor HLA‐B Leader Constellation

Baseline characteristics stratified by the HLA‐B leader constellation are summarised in Table 1. Three hundred and forty‐five of 700 patients (49%) were matched for the HLA‐B leader and 355/700 (51%) had an HLA‐B leader mismatch. Among the 345 HLA‐B leader matched patient/donor pairs, 7 (2%) were MM/MM, 119 (34%) MT/MT and 219 (64%) TT/TT. Among 355 HLA‐B leader mismatched patient/donor pairs, the constellations were as follows: 26 (7%) were MM/MT, 13 (4%) MM/TT, 32 (9%) MT/MM, 121 (34%) MT/TT, 16 (5%) TT/MM and 147 (41%) TT/MT. Significant between‐group differences were observed for recipient age (*p* < 0.001), number of HLA mismatches (*p* < 0.001), and the proportion of recipients with HLA‐B7, −B8 or −B51 alleles (*p* < 0.001).

### 3.3 Recipient HLA‐B Leader and BKPyV Infection

The incidence of decoy cell shedding at 1‐year posttransplant was not different between the three recipient HLA‐B leader groups (MM: 28.3%, MT: 29.0%, TT: 33.5%; log‐rank test *p* = 0.37). The incidence of BKPyV‐DNAemia was also not different between the three groups (MM: 19.6%, MT: 16.5%, TT: 18.9%; log‐rank test *p* = 0.73). In addition, we found no statistically significant differences regarding the BKPyV infection phenotypes (*p* = 0.53) (Figure 2A–C). In the multivariable Cox model, the HLA‐B leader genotype was not associated with decoy cell shedding and BKPyV‐DNAemia (hazard ratio [HR] with 95% confidence interval [CI] given in Figure 2A,B). The HLA‐B leader genotype was also not associated with high BKPyV‐DNAemia > 10′000 GEq/mL (i.e., presumptive BKPyVAN) in a multivariable logistic regression model (*p* = 0.34). Next, we performed the same analyses, but grouped the recipients based on the presence/absence of a −21M peptide in the HLA‐B leader sequences (i.e., MM or MT vs. TT). The incidence of decoy cell shedding at 1‐year posttransplant was not different between the two recipient HLA‐B leader groups (MM or MT: 28.9%, TT: 33.5%; log‐rank test *p* = 0.16). The incidence of BKPyV‐DNAemia was also not different between the two groups (MM or MT: 17.0%, TT: 18.9%; log‐rank test *p* = 0.56). In addition, we found no statistically significant differences regarding the BKPyV infection phenotypes (*p* = 0.54) (Figure 2D–F). Furthermore, in the multivariable Cox model, the HLA‐B leader MM or MT was not associated with decoy cell shedding (HR 0.96 [95% CI: 0.69–1.32], *p* = 0.80; Figure 2D) as well as BKPyV‐DNAemia (HR 1.19 [95% CI: 0.79–1.81], *p* = 0.41; Figure 2E). The HLA‐B leader MM or MT was also not associated with high BKPyV‐DNAemia > 10′000 GEq/mL (i.e., presumptive BKPyVAN) in a multivariable logistic regression model (odds ratio [OR] 1.42 [95% CI: 0.84–2.14], *p* = 0.19).

**FIGURE 2:**
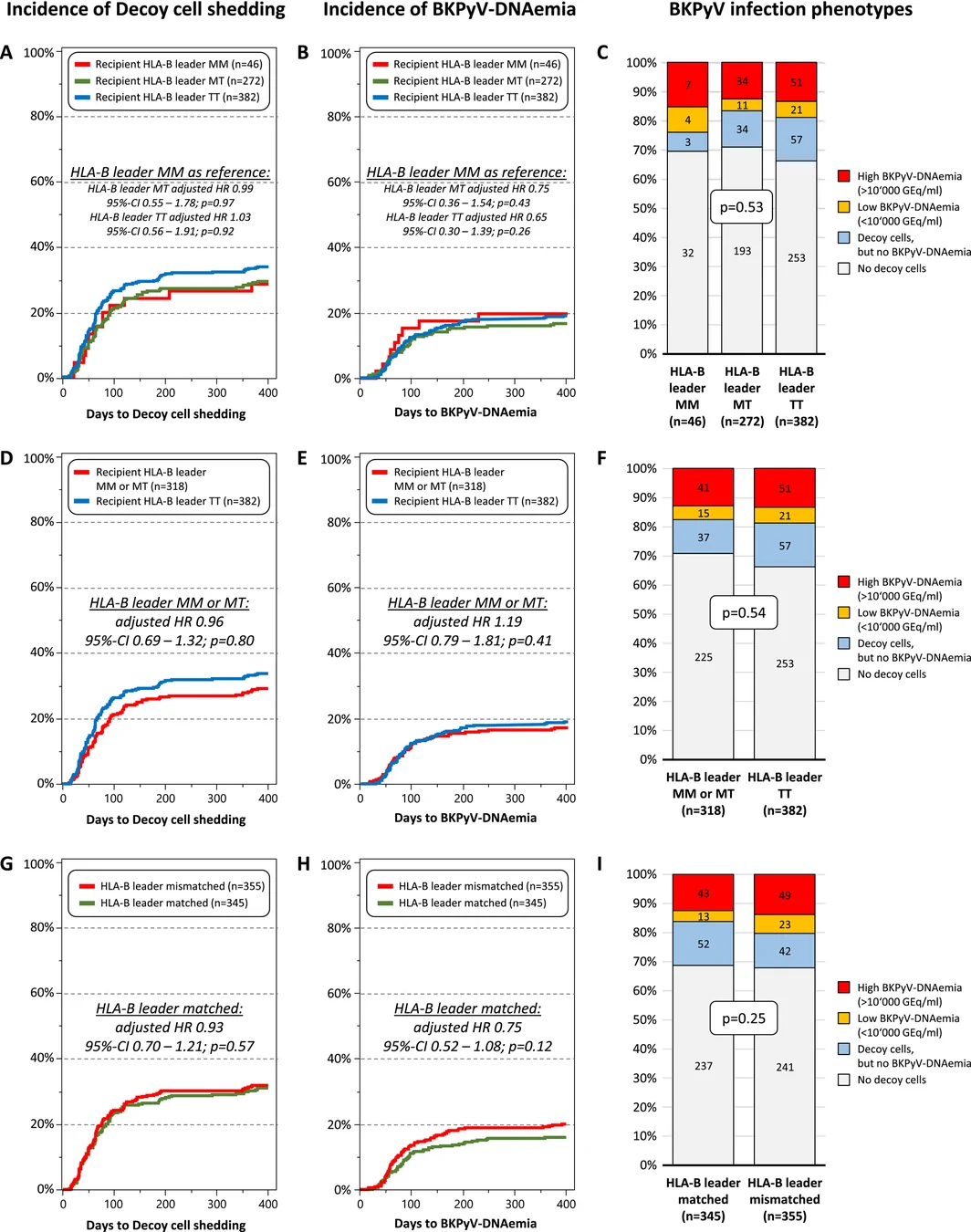
BK polyomavirus time‐to‐event analyses and infection phenotype distribution according to HLA‐B leader constellations. (A, B, C) Stratified by recipient HLA‐B leader genotype (i.e., MM, MT, TT). (D, E, F) Stratified by recipient HLA‐B leader containing a −21M peptide (i.e., MM or MT vs. TT). (G, H, I) Stratified by recipient/donor HLA‐B leader match/mismatch. The adjusted hazard ratios from the multivariable Cox regression models are given in A, B, D, E, G and H.

### 3.4 Recipient/Donor HLA‐B Leader Constellation and BKPyV Infection

The incidence of decoy cell shedding at 1‐year posttransplant was not different between the HLA‐B leader matched and mismatched patients (31.0% vs. 31.8%; log‐rank test *p* = 0.79). The incidence of BKPyV‐DNAemia was also not different between the HLA‐B leader matched and mismatched patients (15.9% vs. 20.0%; log‐rank test *p* = 0.16). In addition, we found no statistically significant differences regarding the BKPyV infection phenotypes between the two groups (*p* = 0.25) (Figure 2G–I). Furthermore, in the multivariable Cox model, the HLA‐B leader constellation was not associated with decoy cell shedding (HR: 0.93 [95% CI 0.70–1.21], *p* = 0.57; Figure 2G; full model shown in Supplementary Table 2) as well as BKPyV‐DNAemia (HR: 0.75 [95% CI: 0.52–1.08], *p* = 0.12; Figure 2H; full model shown in Table 2). The HLA‐B leader constellation was also not associated with high BKPyV‐DNAemia > 10′000 GEq/mL (i.e., presumptive BKPyVAN) in a multivariable logistic regression model (OR 0.88 [95% CI: 0.56–1.38], *p* = 0.58; Supplementary Table 3). Importantly, patients having HLA‐B7, HLA‐B8 or HLA‐B51 alleles were associated with a lower risk of decoy cell shedding (HR 0.74 [95%‐CI 0.56–0.97]; *p* = 0.03; Supplementary Table 2), BKPyV‐DNAemia (HR 0.63 [95% CI: 0.44–0.91]; *p* = 0.01; Table 2), as well as presumptive BKPyVAN (OR 0.58 [95% CI: 0.36–0.91]; *p* = 0.02; Supplementary Table 3).

**TABLE 2:** Multivariable Cox proportional hazards regression model for factors associated with BKPyV‐DNAemia (*n* = 126).

Variable	HR (95% CI)	*p*
HLA‐B leader matched	0.75 (0.52–1.08)	0.12
Recipient age (per decade)	1.04 (0.91–1.18)	0.60
Recipient male sex	1.19 (0.80–1.76)	0.38
ATG induction therapy	0.88 (0.54–1.45)	0.63
HLA‐A/B/C/DRB1/DRB3‐5/DQB1/DPB1 mismatches	1.00 (0.94–1.07)	0.97
Recipient HLA‐B7, HLA‐B8 or HLA‐B51 positive	0.63 (0.44–0.91)	0.01

*Note:* The model was specified to estimate the association between HLA‐B leader matching and BKPyV‐DNAemia. Hazard ratios for the remaining covariates represent adjustment estimates and should not be interpreted as independent causal effects.

Abbreviations: CI, confidence interval; HR, hazard ratio.

Finally, we investigated the association of individual recipient/donor HLA‐B leader constellations with BKPyV infection. We excluded five recipient/donor HLA‐B leader constellations due to low frequencies in our population (MM/MM [*n* = 7; 1.0%], MM/TT [*n* = 13; 1.9%], TT/MM [*n* = 16; 2.3%], MM/MT [*n* = 26; 3.7%] and MT/MM [*n* = 32; 4.6%]), leaving four constellations for the analysis, two matched and two mismatched: MT/MT (*n* = 119; 17.0%), MT/TT (*n* = 121; 17.3%), TT/MT (*n* = 147; 21.0%) and TT/TT (*n* = 219; 31.3%). We observed no difference among these four groups regarding the incidence of decoy cell shedding (log‐rank test *p* = 0.43), the incidence of BKPyV‐DNAemia (log‐rank test *p* = 0.37), and the BKPyV infection phenotypes (*p* = 0.32) (Supplementary Figure 1).

### 3.5 Recipient HLA‐B Leader and CMV Infection

The incidence of CMV‐DNAemia at 1‐year posttransplant was not different between the three recipient HLA‐B leader groups (MM: 19.6%, MT: 30.9%, TT: 28.8%; log‐rank test *p* = 0.35). In addition, we found no statistically significant differences regarding the CMV infection phenotypes (*p* = 0.57) (Figure 3A,B). In the multivariable Cox model, the HLA‐B leader genotype was not associated with CMV‐DNAemia (HR with 95% CI given in Figure 3A). The HLA‐B leader genotype was also not associated with CMV disease in a multivariable logistic regression model (*p* = 0.93). Next, we performed the same analyses, but grouped the recipients based on the presence/absence of a −21M peptide in the HLA‐B leader sequences (i.e., MM or MT vs. TT). The incidence of CMV‐DNAemia at 1‐year posttransplant was not different between the two recipient HLA‐B leader groups (MM or MT: 29.3%, TT: 28.8%; log‐rank test *p* = 0.97). In addition, we found no statistically significant differences regarding the CMV infection phenotypes (*p* = 0.59) (Figure 3C,D). Furthermore, in the multivariable Cox model, the HLA‐B leader MM or MT was not associated with CMV‐DNAemia (HR: 1.15 [95% CI: 0.82–1.61], *p* = 0.42; Figure 3C). The HLA‐B leader MM or MT was also not associated with CMV disease in a multivariable logistic regression model (OR: 0.88 [95% CI: 0.45–1.73], *p* = 0.71).

**FIGURE 3:**
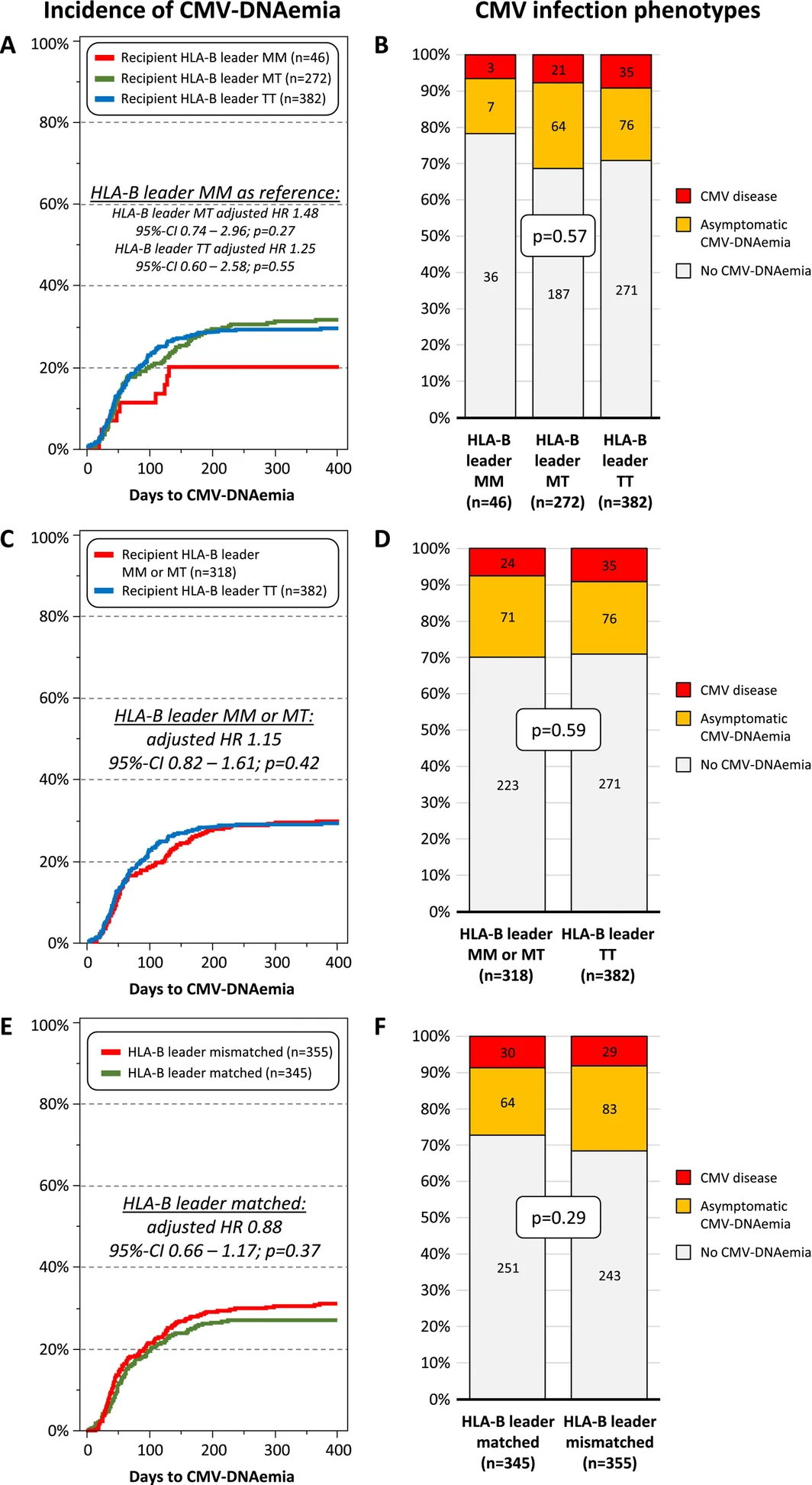
Cytomegalovirus time‐to‐event analysis and infection phenotype distribution according to HLA‐B leader constellation. (A, B) Stratified by recipient HLA‐B leader genotype (i.e., MM, MT, TT). (C, D) Stratified by recipient HLA‐B leader containing a −21M peptide (i.e., MM or MT vs. TT). (E, F) Stratified by recipient/donor HLA‐B leader match/mismatch. The adjusted hazard ratios from the multivariable Cox regression models are given in A, C and E.

### 3.6 Recipient/Donor HLA‐B Leader Constellation and CMV Infection

The incidence of CMV‐DNAemia at 1‐year posttransplant was not different between the HLA‐B leader matched and mismatched patients (27.0% vs. 31.0%; log‐rank test *p* = 0.25). In addition, we found no statistically significant differences regarding the CMV infection phenotypes between the two groups (*p* = 0.29) (Figure 3E,F). Furthermore, in the multivariable Cox model, recipient age (HR 1.16 [95% CI: 1.04–1.29], *p* = 0.007) and CMV serostatus (*p* < 0.001) were significantly associated with CMV‐DNAemia, but the HLA‐B leader constellation was not (HR 0.88 [95% CI: 0.66–1.17], *p* = 0.37; Figure 3E; full model shown in Table 3). The HLA‐B leader was also not associated with CMV disease in a multivariable logistic regression model (OR 1.10 [95% CI: 0.62–1.93], *p* = 0.75; Supplementary Table 4). However, CMV serostatus remained significantly associated with CMV disease (low risk as reference; intermediate risk OR 8.23 [95% CI: 1.92–35.19]; high risk OR 33.69 [95% CI: 6.11–185.79], *p* < 0.001; Supplementary Table 4). We observed no difference between the HLA‐B leader matched/mismatched groups regarding the subgroups of CMV disease (i.e., CMV syndrome vs. tissue‐invasive CMV disease; *p* = 0.12; data not shown).

**TABLE 3:** Multivariable Cox proportional hazards regression model for factors associated with CMV‐DNAemia (*n* = 203).

Variable	HR (95% CI)	*p*
HLA‐B leader matched	0.88 (0.66–1.17)	0.37
Recipient age (per decade)	1.16 (1.04–1.29)	0.007
HLA‐A/B/C/DRB1/DRB3‐5/DQB1/DPB1 mismatches	1.05 (0.99–1.11)	0.10
Recipient HLA‐B7, HLA‐B8 or HLA‐B51 positive	0.93 (0.70–1.23)	0.62
CMV prophylaxis with Valganciclovir	^a^	
CMV serostatus		< 0.001
Low risk (D−/R−)	Reference	
Intermediate risk (R+)	30.11 (9.56–94.82)	
High risk (D+/R−)	47.10 (13.78–161.03)	

*Note:* The model was specified to estimate the association between HLA‐B leader matching and CMV‐DNAemia. Hazard ratios for the remaining covariates represent adjustment estimates and should not be interpreted as independent causal effects.

Abbreviations: CI, confidence interval; HR, hazard ratio.

^a^ CMV prophylaxis was stratified due to violation of the proportional hazards assumption.

Finally, we investigated the association of the four most frequent individual recipient/donor HLA‐B leader constellations as described above with CMV infection. We observed no difference among these four groups regarding the incidence of CMV‐DNAemia (log‐rank test *p* = 0.56) and the CMV infection phenotypes (*p* = 0.48) (Supplementary Figure 2).

## 4 Discussion

The major finding of this study was that neither occurrence nor severity of CMV and BKPyV infection in the first year after renal transplantation were associated with the recipient HLA‐B leader genotype and the recipient/donor HLA‐B leader constellation. These findings confirm the observation by Aubry et al. [20] regarding CMV‐DNAemia, but stand in contrast to their data reporting a strong protective effect of an HLA‐B leader mismatch against BKPyV‐DNAemia.

The two cohorts differ in size (*n* = 306 vs. *n* = 700) and the proportion of living donor transplants (8% vs. 41%) but were very similar regarding the frequency of HLA‐B leader genotypes of the recipients and donors as well as the used immunosuppressive regimens. The classification as CMV‐DNAemia and BKPyV‐DNAemia was slightly different due to various assay thresholds for positivity. Aubry defined patients as ‘BKPyV positive’, if BKPyV‐DNAemia was > 200 c/mL or BKPyV‐DNAuria > 107 c/mL, whereas we used a threshold for BKPyV‐DNAemia positivity of > 1000 GEq/mL. Notably, the overall incidence of such defined BKPyV‐events was similar in both studies (23% vs. 18%). By contract, the incidence of CMV‐DNAemia was higher in the study by Aubry (40% vs. 29%). We can only speculate why the association of BKPyV‐DNAemia and the HLA‐B leader mismatch was not confirmed in our study, which might partially be related to the larger population size.

The main reason for this discrepancy probably lies in the complex interaction of various factors influencing susceptibility for viral infections in kidney transplant recipients. Besides the level of immunosuppression, viral pathogenicity factors, CD8+ cytotoxic T‐cell and NK cell function, HLA phenotypes of the recipient and donor, NK cell receptors and their ligands on cell surfaces as well as HLA‐E polymorphism and expression level could contribute to viral infection control [26, 27, 28]. Interestingly, it has been proposed that −21M leader peptides contribute to antiviral response more effectively than −21T leader peptides, as they bind HLA‐E more efficiently and lead to a higher HLA‐E cell surface expression which might lead to a better education of NK cells [16]. However, Lin et al. [29] demonstrated that the −21M leader peptide, despite leading to higher HLA‐E cell surface expression, is a poor ligand for CD94/NKG2A and, by competing with other ligands with better receptor recognition, could reduce CD94/NKG2A recognition of target cells. This illustrates the complex relationship between HLA‐B leader peptides and antiviral protection.

Consistent with previous reports, recipient HLA‐B7 and/or HLA‐B8 and/or HLA‐B51 antigens seem to be associated with a lower risk of BKPyV infection after kidney transplantation [11, 12]. This effect was observed for decoy cell shedding as a surrogate for high‐level BKPyV‐DNAuria, for new‐onset of BKPyV‐DNAemia as well as for presumptive BKPyVAN. However, the calculated risk reduction for BKPyV‐DNAemia was only 37%, which would translate into a reduction of the 1‐year incidence of BKPyV‐DNAemia from 18% to 11%. While conceptually important, this is not sufficient to selectively adapt the BKPyV screening or management procedures for such patients. Interestingly, recipient HLA‐B7 and/or HLA‐B8, and/or HLA‐B51 antigens were not protective against CMV infections suggesting that this effect is specific for BKPyV.

The strengths of this study are the cohort size (*n* = 700), the accurate determination of the HLA‐B leader dimorphism enabled by high‐resolution HLA‐typing of the recipients and the donors, the granular assessment of BKPyV and CMV infections, and the uniform maintenance immunosuppression based on Tac‐MPA‐P.

We note the following limitations. First, this is a single centre retrospective analysis of prospectively collected data in an almost exclusively Caucasian population. Therefore, our results might not be fully transferable to other kidney transplant programmes with different patient ethnicities. Furthermore, the results are only applicable to patients with a Tac‐MPA‐P maintenance immunosuppression having either basiliximab or ATG induction. However, this immunosuppressive regimen can be considered as a standard therapy, which is very widely used. In addition, the study population might still not be large enough to detect small effects of the HLA‐B leader status on BPKyV or CMV infections, but even if such effects exist, they are alone not likely to support a change in current viral screening strategies. Finally, HLA‐E typing data were not available, which might have been informative.

In conclusion, the recipient HLA‐B leader genotype and the recipient/donor HLA‐B leader constellation are neither associated with the occurrence nor the severity of BKPyV and CMV infections within the first year posttransplant in kidney allograft recipients. This argues against the use of HLA‐B leader information to guide current screening and management strategies for BKPyV and CMV.

## Author Contributions

Participated in research design: L.B., M.D., S.S. Participated in the writing of the paper: all. Participated in the performance of the research: all. Participated in data analysis: L.B., M.D., S.S.

## Funding

The authors have nothing to report.

## Ethics Statement

This study was approved by the local ethics committee of North‐Western and Central Switzerland (EKNZ 2026–00962).

## Conflicts of Interest

The authors declare no conflicts of interest.

## Supporting information


**Table 1:** Baseline characteristics of patients according to recipient HLA‐B leader.
**Table 2:** Multivariable Cox proportional hazards regression model for factors associated with decoy cell shedding (*n* = 220).
**Table 3:** Multivariable binary logistic regression model for factors associated with high BKPyV‐DNAemia > 10′000 GEq/mL (i.e., presumptive BKPyVAN; *n* = 92).
**Table 4:** Multivariable binary logistic regression model for factors associated with CMV disease (*n* = 59).

**Supplementary Figure 1:** BK polyomavirus time‐to‐event analyses and infection phenotype distribution according to individual recipient(R)/donor(D) HLA‐B leader constellations.
**Supplementary Figure 2:** CMV time‐to‐event analyses and infection phenotype distribution according to individual recipient(R)/donor(D) HLA‐B leader constellations.

## Data Availability

The data that support the findings of this study are available on request from the corresponding author. The data are not publicly available due to privacy or ethical restrictions.
