# Supplementary material for: Association of HLA‐B Leader With BK Polyomavirus and Cytomegalovirus Infections in Kidney Allograft Recipients
Source: HLA. 2026 Nov 6;108(5):e71082. doi: 10.1111/tan.71082 (PMC13741198; doi:10.1111/tan.71082)
Supplement: Supplementary file 1 [file TAN-108-e71082-s001.docx]

**Supplementary tables**

**Table 1. Baseline characteristics of patients according to recipient HLA-B leader.**

| **Variable** | **Whole population**  **(n=700)** | **HLA-B leader MM or MT**  **(n=318)** | **HLA-B leader TT**  **(n=382)** | **p-value** |
| --- | --- | --- | --- | --- |
| Recipient age (years) | 55 (44-63) | 56 (46-63) | 55(43-63) | 0.27 |
| Recipient female, n (%) | 240 (34) | 113 (36) | 127 (33) | 0.53 |
| Underlying nephropathy, n (%) |  |  |  | 0.13 |
| Glomerulopathy | 234 (33) | 109 (34) | 113 (32) |  |
| Vascular/hypertensive | 67 (10) | 32 (10) | 35 (9) |  |
| Diabetic | 55 (8) | 20 (6) | 35 (9) |  |
| ADPKD | 144 (21) | 71 (22) | 73 (19) |  |
| Interstitial | 38 (5) | 12 (4) | 26 (7) |  |
| Unknown | 64 (9) | 23 (7) | 41 (11) |  |
| Other | 98 (14) | 51 (16) | 47 (12) |  |
| Induction therapy, n (%) |  |  |  | 0.31 |
| ATG | 123 (18) | 61 (19) | 62 (16) |  |
| Basiliximab | 577 (82) | 257 (81) | 320 (84) |  |
| Living donor, n (%) | 290 (41) | 135 (42) | 155 (41) | 0.62 |
| Donor age (years) | 56 (47-64) | 57(47-64) | 56 (47-63) | 0.67 |
| First kidney transplant, n (%) | 605 (86) | 268 (84) | 337 (88) | 0.13 |
| CMV serostatus, n (%) |  |  |  | 0.55 |
| Low risk (D-/R-) | 170 (24) | 80 (25) | 90 (24) |  |
| Intermediate risk (R+) | 409 (58) | 179 (56) | 230 (60) |  |
| High risk (D+/R-) | 121 (17) | 59 (19) | 62 (16) |  |
| CMV prophylaxis with Valganciclovir, n (%) | 243 (35) | 123 (39) | 120 (31) | 0.04 |
| Pretransplant HLA-DSA, n (%) | 91 (13) | 46 (14) | 45 (12) | 0.29 |
| HLA-A/B/C/DRB1/DRB3-5/DQB1/DPB1 mismatches* | 8 (6-10) | 7 (5-9) | 8 (6-10) | 0.02 |
| Recipient HLA-B7, HLA-B8 or HLA-B51 positive, n (%) | 338 (48) | 249 (78) | 89 (23) | <0.001 |
| ABO incompatible, n (%) | 55 (8) | 26 (8) | 29 (8) | 0.77 |
| Delayed graft function, n (%) | 155 (22) | 60 (19) | 95 (25) | 0.06 |

* HLA mismatches are calculated at the level of serological equivalents

Abbreviations: ADPKD = autosomal dominant polycystic kidney disease; ATG = anti-thymocyte globulin; CMV = Cytomegalovirus; HLA-DSA = donor-specific HLA-antibodies

**Table 2. Multivariable Cox proportional hazards regression model for factors associated with decoy cell shedding (n=220).**

| **Variable** | **HR (95% CI)** | **p-value** |
| --- | --- | --- |
| HLA-B leader matched | 0.93 (0.70-1.21) | 0.57 |
| Recipient age (per decade) | 1.02 (0.93-1.13) | 0.67 |
| Recipient male sex | 1.14 (0.85-1.53) | 0.38 |
| ATG induction therapy | 0.67 (0.45-1.00) | 0.05 |
| HLA-A/B/C/DRB1/DRB3-5/DQB1/DPB1 mismatches | 0.99 (0.94-1.04) | 0.56 |
| Recipient HLA-B7, HLA-B8, or HLA-B51 positive | 0.74 (0.56-0.97) | 0.03 |

Abbreviations: HR = Hazard Ratio ; CI = Confidence Interval. The model was specified to estimate the association between HLA-B leader matching and decoy cell shedding. Hazard ratios for the remaining covariates represent adjustment estimates and should not be interpreted as independent causal effects.

**Table 3. Multivariable binary logistic regression model for factors associated with** **high BKPyV-DNAemia >10'000 GEq/ml (i.e. presumptive BKPyVAN; n=92).**

| **Variable** | **OR (95% CI)** | **p-value** |
| --- | --- | --- |
| HLA-B leader matched | 0.88 (0.56-1.38) | 0.58 |
| Recipient age (per decade) | 1.09 (0.93-1.30) | 0.29 |
| Recipient male sex | 1.44 (0.88-2.43) | 0.15 |
| ATG induction therapy | 1.01 (0.53-1.83) | 0.98 |
| HLA-A/B/C/DRB1/DRB3-5/DQB1/DPB1 mismatches | 1.02 (0.93-1.11) | 0.71 |
| Recipient HLA-B7, HLA-B8, or HLA-B51 positive | 0.58 (0.36-0.91) | 0.02 |

Abbreviations: OR = Odds Ratio; CI = Confidence Interval. The model was specified to estimate the association between HLA-B leader matching and high BKPyV-DNAemia >10'000 GEq/ml. Odds ratios for the remaining covariates represent adjustment estimates and should not be interpreted as independent causal effects.

**Table 4. Multivariable binary logistic regression model for factors associated with CMV disease (n=59).**

| **Variable** | **OR (95% CI)** | **p-value** |
| --- | --- | --- |
| HLA-B leader matched | 1.10 (0.62-1.93) | 0.75 |
| Recipient age (per decade) | 1.13 (0.92-1.40) | 0.25 |
| HLA-A/B/C/DRB1/DRB3-5/DQB1/DPB1 mismatches | 1.03 (0.92-1.15) | 0.62 |
| Recipient HLA-B7, HLA-B8, or HLA-B51 positive | 0.80 (0.46-1.40) | 0.44 |
| CMV prophylaxis with Valganciclovir | 0.64 (0.27-1.52) | 0.31 |
| CMV serostatus |  | <0.001 |
| Low risk (D-/R-) | reference |  |
| Intermediate risk (R+) | 8.23 (1.92-35.19) |  |
| High risk (D+/R-) | 33.69 (6.11-185.79) |  |

Abbreviations: OR = Odds Ratio; CI = Confidence Interval. The model was specified to estimate the association between HLA-B leader matching and CMV disease. Odds ratios for the remaining covariates represent adjustment estimates and should not be interpreted as independent causal effects.
